# Goblet Cell-Mediated Pathway: A Major Contributor to Increased Intestinal Permeability in Streptozotocin-Induced Type 1 Diabetic Mice

**DOI:** 10.3390/ijms26188890

**Published:** 2025-09-12

**Authors:** Ming-Hsun Wu, Lee-Wei Chen, Jiann-Hwa Chen, Chieh-Wen Lai

**Affiliations:** 1 Department of Surgery, National Taiwan University Hospital, Taipei 100229, Taiwan; 2 Department of Surgery, Kaohsiung Veterans General Hospital, Kaohsiung 813414, Taiwan; 3 Department of Gastroenterology, Taipei Tzu Chi Hospital, Buddhist Tzu Chi Medical Foundation, New Taipei City 231405, Taiwan; 4 School of Medicine, Tzu Chi University, Hualien 970374, Taiwan; 5 Department of Surgery, Taipei Tzu Chi Hospital, Buddhist Tzu Chi Medical Foundation, New Taipei City 231405, Taiwan

**Keywords:** intravital multiphoton microscopy, intestinal permeability, goblet cells, type 1 diabetes mellitus, fructooligosaccharide

## Abstract

Gut barrier dysfunction and increased intestinal permeability are closely linked to the pathogenesis of type 1 diabetes and its complications. Streptozotocin (STZ)-induced diabetic mice, which mimic β-cell destruction and insulin deficiency, provide a widely used model for studying type 1 diabetes-associated intestinal barrier impairment. However, the cellular pathways mediating this dysfunction, particularly the role of goblet cells, remain incompletely elucidated. This study aimed to investigate the association between the gut barrier function and diabetes. Using real-time intravital multiphoton microscopy, we investigated intestinal barrier integrity in STZ-induced type 1 diabetic mice. Three groups were analysed: the control, STZ-diabetic, and STZ-diabetic mice treated with fructooligosaccharide (FOS) for 1 week. Intestinal permeability was assessed by measuring fluorescein isothiocyanate (FITC)-dextran concentrations in the portal vein and visualising translocation into villi. Epithelial morphology was examined, focusing on goblet cell density and leakage pathways. STZ-diabetic mice demonstrated a significant increase in intestinal permeability, evidenced by elevated FITC-dextran levels in the portal vein and villi. Multiphoton imaging revealed a notable rise in the goblet cell-to-enterocyte ratio in diabetic mice, while the gap density remained unchanged. The predominant route of macromolecular leakage in STZ-diabetic mice was via goblet cells rather than by paracellular gaps. One-week FOS supplementation significantly reduced goblet cell density and partially restored barrier function without altering the distribution of leakage pathways. These findings highlight goblet cell-mediated transcellular leakage as a major mechanism of gut barrier dysfunction in type 1 diabetic mice. Short-term FOS treatment partially reverses these alterations. Targeting goblet cell function may offer a promising therapeutic strategy to restore gut barrier integrity in diabetes.

## 1. Introduction

The human intestinal epithelium serves a critical dual function: it facilitates nutrient and water absorption and acts as a selective barrier against luminal pathogens, toxins, and pro-inflammatory mediators. The disruption of this barrier permits the translocation of microbial components and dietary antigens into the systemic circulation, thereby contributing to the pathogenesis of inflammatory disorders, infections, and sepsis [1,2,3].

Anatomically, the intestinal barrier comprises a single layer of epithelial cells interconnected by tight junctions and is supported by a mucus layer and mucosal immune components. Its integrity is maintained through a tightly regulated balance between cellular apoptosis and regeneration, primarily driven by stem cell migration from intestinal crypts toward the villus tips. In addition to their absorptive function, epithelial cells play an immunological role by recognising microbial stimuli and modulating innate and adaptive immune responses [4,5]. Compromised barrier integrity leads to increased intestinal permeability, enabling luminal antigens and microorganisms to trigger systemic immune activation [6].

Recent studies have highlighted the intricate interplay between type 1 diabetes and gut barrier dysfunction. In type 1 diabetes, autoimmune-mediated β-cell destruction leads to insulin deficiency, which has been associated with increased intestinal permeability and systemic inflammation. Individuals with type 1 diabetes frequently exhibit these alterations, which may facilitate microbial translocation and metabolic dysregulation [7,8,9]. Moreover, low-grade inflammation and microbiota alterations frequently observed in patients with diabetes may further compromise the gut barrier, establishing a bidirectional cycle of disease progression [10,11].

Although substantial in vitro and ex vivo evidence has elucidated the cellular and molecular mechanisms linking diabetes to gut barrier impairment [12,13,14], in vivo studies remain limited. Advances in intravital imaging techniques, such as multiphoton microscopy, have enabled the direct visualisation of intestinal permeability in live animals. In our previous study using a live mouse model, we demonstrated that endotoxemia induces gut barrier dysfunction through increased epithelial shedding and sustained gap formation [15]. Nevertheless, real-time in vivo data depicting morphological alterations of the intestinal epithelium in diabetic models, particularly under inflammatory stress, remain scarce. In addition, most studies have focused predominantly on the paracellular permeability pathway [16,17,18], underscoring the need for more comprehensive in vivo evaluations.

Restoring gut barrier integrity has emerged as a potential therapeutic target in metabolic diseases [19]. Among various interventions, prebiotics—non-digestible dietary fibres that selectively stimulate the growth of beneficial gut microbiota—have shown promise in modulating the intestinal barrier function [20,21]. By enhancing microbial diversity and suppressing inflammation, prebiotics help reinforce epithelial integrity and reduce permeability. Several studies have reported that prebiotic supplementation not only attenuates intestinal inflammation but also improves metabolic outcomes in animal models of diabetes [22,23,24].

Given the central role of the gut barrier in systemic homeostasis and metabolic regulation, further in vivo studies are needed. Therefore, the present study aimed to investigate intestinal barrier dysfunction in streptozotocin (STZ)-induced type 1 diabetic mice, with a specific focus on goblet cell dynamics and epithelial gap density and to evaluate whether short-term prebiotic intervention with fructooligosaccharides (FOS) could restore barrier integrity. In this study, we employed streptozotocin (STZ)-induced diabetic mice, a well-established animal model of type 1 diabetes characterised by selective β-cell destruction and insulin deficiency. This model was chosen because it reliably reproduces the hyperglycaemic and inflammatory conditions that contribute to gut barrier dysfunction in type 1 diabetes, thereby allowing the investigation of underlying epithelial mechanisms and potential interventions.

## 2. Results

### 2.1. Real-Time Dynamic Changes in Goblet Cells and Epithelial Cell Shedding

**Figure 1:**
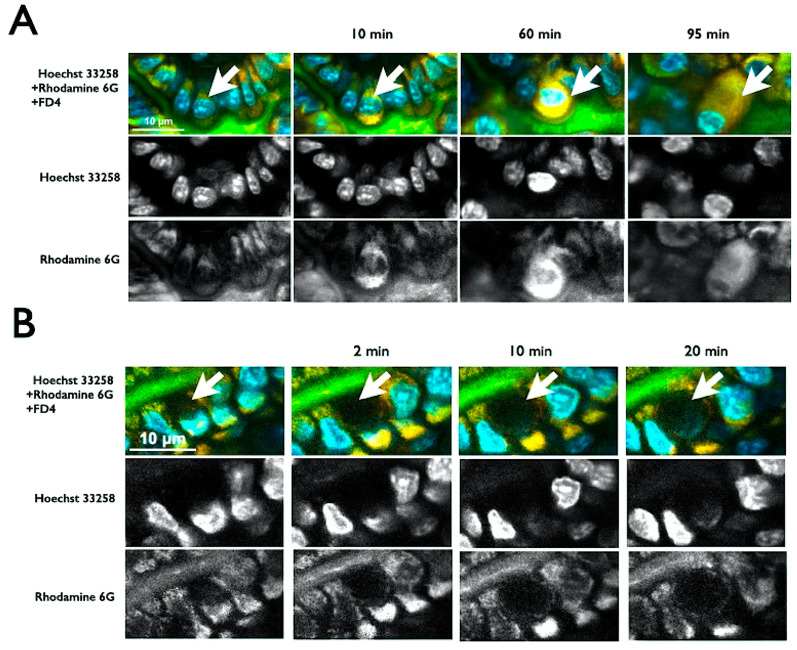
Real-time imaging of epithelial cell shedding and goblet cell dynamics in the intestinal mucosa. (**A**) Time-lapse multiphoton images show epithelial cell shedding, where shedding cells exhibit increased cytoplasmic Rhodamine 6G signal before detachment, forming a cell-free gap negative for Hoechst 33258 and Rhodamine 6G (arrows). (**B**) Goblet cells remain positive for both stains, with their cytoplasm gradually enlarging and the nucleus displaced toward the basal side over time (arrows). Scale bar: 10 μm.

Using multiphoton microscopy, we visualised epithelial dynamics in the control and STZ-diabetic mice by staining nuclei with Hoechst 33258 and cytoplasm with Rhodamine 6G, while bathing the lumen with FD4. The process of epithelial cell shedding is shown in Figure 1A. At the onset of shedding, an increase in the Rhodamine 6G fluorescence intensity was observed in the cytoplasm. As the process continued, condensed cytoplasmic material detached from neighbouring cells and was released into the intestinal lumen, ultimately resulting in a cell-free gap, which was negative for Hoechst 33258 and Rhodamine 6G staining (arrow, Figure 1A).

In contrast, goblet cells remained distinctly positive for nuclear and cytoplasmic stains. Their cytoplasm, containing mucinogen granules, progressively enlarged in time-lapse imaging, eventually displacing the nucleus toward the basal pole (arrow, Figure 1B). These features allowed for the clear identification of goblet cells and cell-shedding events using intravital multiphoton microscopy.

### 2.2. Impact of STZ and FOS on Goblet Cell and Gap Densities

(a) STZ-induced type 1 diabetes increases the goblet cell density but not gap density.

Representative intravital multiphoton images of the intestinal epithelium from the control, STZ, and STZ + FOS groups are shown below. Nuclei were stained with Hoechst 33258 (blue), cytoplasm with Rhodamine 6G (yellow), and the intestinal lumen was bathed with FD4 (green). White boxed regions are shown at a higher magnification below (a–c). Cell-free gaps indicate sites of epithelial cell shedding, while goblet cells (arrows) are identifiable by their distinct morphology and intense cytoplasmic staining.

**Figure 2:**
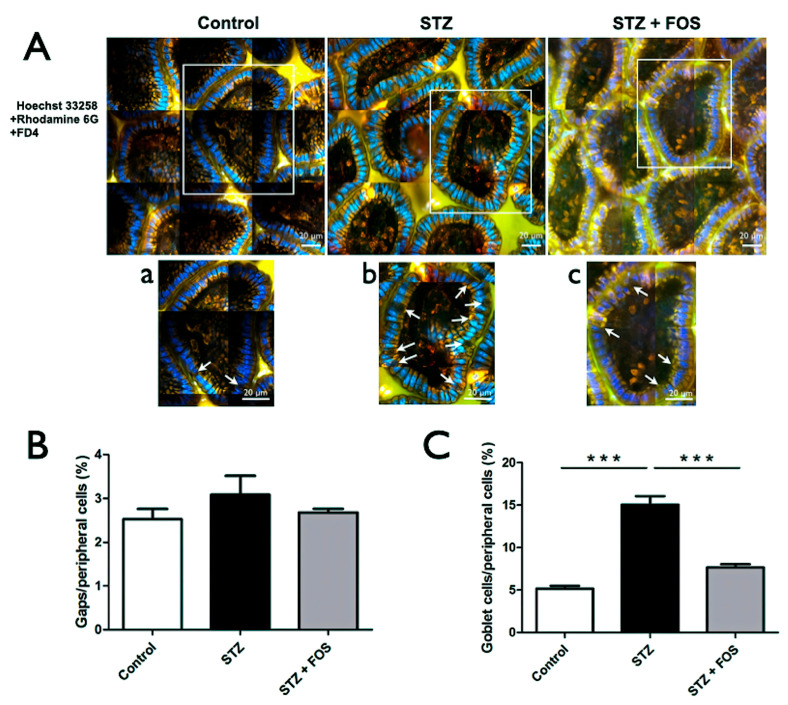
Effects of STZ and FOS on intestinal epithelial morphology, gap formation, and goblet cell ratio. (**A**) Representative multiphoton images of intestinal villi from the control, STZ, and STZ+FOS groups, stained with Hoechst 33258 (nuclei, blue), Rhodamine 6G (cytoplasm, yellow), and FD4 (green). Insets (a–c) highlight cell-free gaps and goblet cells (arrows). (**B**) Quantification of cell-free gaps as a percentage of peripheral cells. (**C**) Quantification of goblet cells as a percentage of peripheral cells; STZ increased the goblet cell proportion, which was reduced by FOS supplementation. *** *p* < 0.001. Scale bar: 20 μm. STZ, Streptozotocin; FOS, fructooligosaccharide.

A quantitative analysis was performed in all three groups (control, STZ, and STZ + FOS), and it was revealed that the proportion of goblet cells among peripheral epithelial cells in the intestinal villi was significantly higher in the STZ group than in the controls (15.04 ± 1.75% vs. 5.15 ± 0.34%, *p* < 0.05; Figure 2C). However, the density of gaps between peripheral cells was not significantly different between groups (3.08 ± 0.73% vs. 2.52 ± 0.23%; Figure 2B).

(b) FOS supplementation reverses STZ-induced increases in the goblet cell density.

After 1 week of FOS treatment in STZ-induced type 1 diabetic mice, the ratio of goblet cells to peripheral cells was significantly decreased compared to that in the untreated STZ group (7.65 ± 0.65% vs. 15.04 ± 1.75%, *p* < 0.05; Figure 2C), whereas the gap density remained unchanged (Figure 2B). These results suggest that FOS supplementation specifically restores goblet cell density without affecting epithelial gap formation.

### 2.3. Effects of STZ and FOS on Intestinal Permeability

(a) STZ-induced type 1 diabetes increases intestinal permeability.

**Figure 3:**
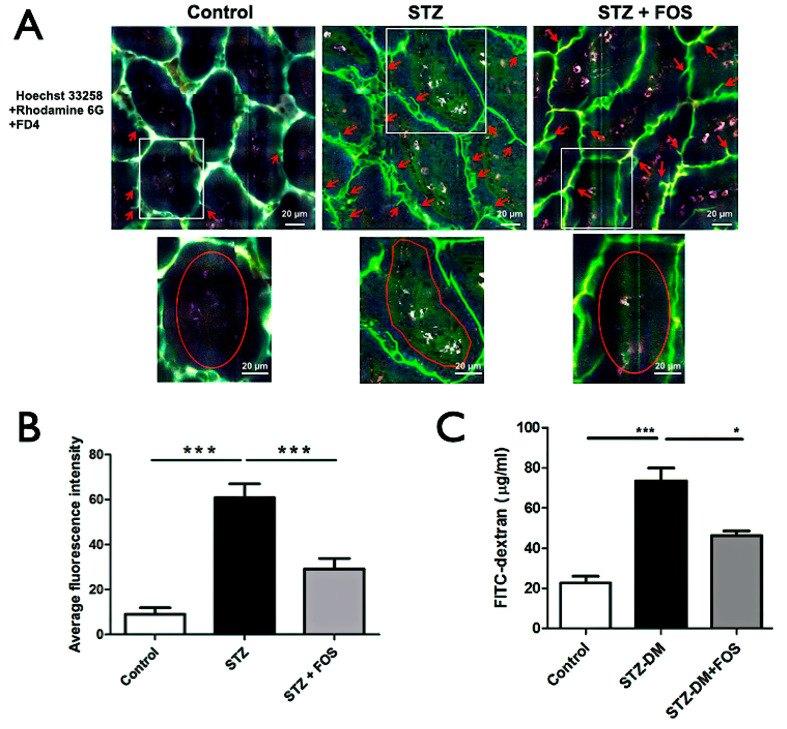
STZ-induced type 1 diabetes increases intestinal permeability, reversed by FOS supplementation. (**A**) Multiphoton images show FD4 leakage (red arrows) from the lumen into central villi in control, STZ, and STZ + FOS groups. White boxes indicate the regions of interest that were magnified in the insets for quantification. Red circles highlight representative areas where FD4 penetration is evident within villi. Insets highlight regions used for quantification. (**B**) Quantification of central villi fluorescence intensity using multiphoton autofluorescence intensity (MAFI), which reflects the degree of FD4 penetration into the villi. STZ significantly increases MAFI, indicating higher permeability, while FOS supplementation reduces MAFI. (**C**) Portal vein FITC-dextran levels. STZ elevates intestinal permeability, partially restored by FOS. *** *p* < 0.001, * *p* < 0.05. Scale bar: 20 μm.

FITC-dextran concentrations in portal vein blood were significantly higher in STZ-induced type 1 diabetic mice than in the controls (Figure 3C). In addition, multiphoton imaging demonstrated the prominent protrusion of FD4 from the intestinal lumen into the central villi in the STZ group (red arrow, Figure 3A). Quantitatively, the multiphoton autofluorescence intensity (MAFI) within the central villus region was markedly elevated in STZ mice (60.81 ± 6.17) than in the controls (9.03 ± 2.85, *p* < 0.05; Figure 3B), confirming the increased intestinal permeability.

(b) FOS treatment restores intestinal permeability.

After 1 week of FOS supplementation, FITC-dextran concentrations in the portal vein of STZ-induced type 1 diabetic mice were significantly reduced compared to those in the untreated STZ mice (Figure 3C). Similarly, MAFI was significantly lower in the STZ + FOS group than in the STZ group (29.12 ± 4.74 vs. 60.81 ± 6.17, *p* < 0.05; Figure 3B), indicating the partial restoration of intestinal barrier function.

### 2.4. Goblet Cells as the Primary Leak Pathway in STZ-Induced Type 1 Diabetes

**Figure 4:**
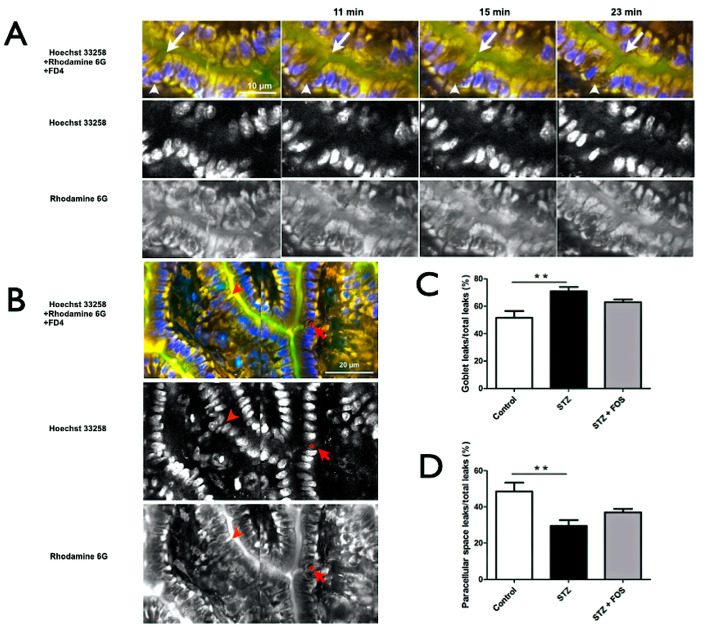
Goblet cells as the predominant leak pathway in STZ-induced diabetic mice. (**A**) Time-lapse multiphoton images showing the dynamic entry of FD4 (green) from the intestinal lumen into goblet cells (arrows) in vivo. Nuclei are stained with Hoechst 33258 (blue), cytoplasm with Rhodamine 6G (yellow), and FD4 marks leak pathways. Merged and single-channel images are shown at 11, 15, and 23 min, with arrowheads indicating paracellular leak sites. (**B**) Representative images illustrating two distinct leak pathways: FD4 entry through goblet cells (arrows and stars indicate goblet cells) and paracellular leaks (arrowheads). (**C**) Quantification of goblet cell-mediated leaks, expressed as a percentage of total leak events. STZ-induced diabetic mice show a significantly higher proportion of goblet cell leaks than the controls, with no significant reduction following FOS supplementation. (**D**) Quantification of paracellular leaks as a percentage of total leak events. STZ significantly reduces the proportion of paracellular leaks versus controls; FOS supplementation does not significantly alter this distribution. Scale bars: 10 μm (**A**), 20 μm (**B**). Statistical significance: ** *p* < 0.01. STZ, Streptozotocin; FOS, fructooligosaccharide.

Leak pathways were further examined using FD4 in the intestinal lumen. Time-lapse imaging (Figure 4A) demonstrated progressive FD4 fluorescence (green, arrow) in goblet cells, which stained positively with Hoechst 33258 (blue) and Rhodamine 6G (yellow). Figure 4B illustrates paracellular leaks (arrowhead) and leaks through goblet cells (arrow; star indicates goblet cell). The proportion of leaks via goblet cells was significantly higher in STZ mice than in the controls (70.93 ± 5.33% vs. 51.58 ± 4.95%, *p* < 0.05; Figure 4C), whereas paracellular leaks were significantly lower (29.39 ± 5.52% vs. 48.42 ± 4.95%, *p* < 0.05; Figure 4D). FOS treatment did not significantly affect the distribution of leak pathways (Figure 4C,D), suggesting that goblet cells are the predominant leak pathway in STZ-induced type 1 diabetic mice.

## 3. Discussion

This study used real-time intravital multiphoton microscopy to elucidate the dynamic morphological and functional changes in the intestinal epithelium under type 1 diabetic conditions and explore the therapeutic potential of FOS in restoring the gut barrier function. Our findings provide novel insights into the cellular mechanisms underlying increased intestinal permeability in type 1 diabetes and highlight the key role of goblet cells in this process.

Consistent with previous studies, we observed a significant increase in intestinal permeability in STZ-induced type 1 diabetic mice, as evidenced by elevated FITC-dextran concentrations in the portal vein and enhanced FITC signals within the central villi [25,26]. Real-time imaging further revealed that this increased permeability was accompanied by a substantial rise in the ratio of goblet cells to surface villus enterocytes, while the density of epithelial gaps remained unchanged. These findings suggest that goblet cell hyperplasia may contribute more critically to gut barrier dysfunction in the type 1 diabetic state rather than enhanced cell shedding or gap formation.

A major novel observation in our study is the identification of goblet cells as the primary route for macromolecular leakage across the intestinal epithelium in type 1 diabetic mice. Time-lapse imaging demonstrated that a significantly greater proportion of FITC-dextran leakage occurred through goblet cells in the STZ group than in the controls, whereas the paracellular (gap-mediated) leakage pathway was less prominent. This contrasts with our previous findings in sepsis models, where paracellular leakage was predominant [15]. This divergence emphasises that the dominant leak pathway is disease-specific, with hyperglycaemia and chronic inflammation in type 1 diabetes driving goblet cell-mediated leakage, while acute systemic inflammation in sepsis promotes paracellular disruption.

Goblet cells are classically known as mucin-secreting cells essential for maintaining the mucus barrier; however, accumulating evidence suggests they also serve as active participants in antigen transport and immune regulation [27,28,29]. Our in vivo observations reinforce this emerging concept, indicating that goblet cell hyperplasia in type 1 diabetes may create a double-edged effect: while maintaining mucus secretion, it simultaneously facilitates luminal antigen passage, thereby fuelling systemic immune activation and low-grade inflammation. This mechanism could help explain the heightened susceptibility of patients with type 1 diabetes to infections and inflammatory complications, as reported in clinical studies [30,31,32,33].

Importantly, we demonstrated that the short-term administration of FOS to type 1 diabetic mice significantly reduced goblet cell density and partially restored gut barrier function, as indicated by the decreased FITC-dextran permeability and improved villus morphology. Notably, FOS treatment did not alter the distribution of leakage pathways, suggesting that its main effect is mediated through the normalisation of goblet cell dynamics rather than by restoring paracellular integrity. This aligns with previous reports showing that prebiotics promote short-chain fatty acid (SCFA) production, which in turn stimulates mucin secretion, enhances epithelial renewal, and exerts anti-inflammatory effects [22]. Moreover, microbiota-derived SCFAs, which have been reported to regulate goblet cell mucin secretion and promote regulatory T-cell differentiation, may provide a mechanistic link between epithelial barrier function and systemic immune regulation [34]. Thus, our study provides in vivo evidence supporting a microbiota–SCFA–goblet cell axis as a plausible mechanism by which FOS improves the gut barrier function.

Our study has several implications. First, it establishes a direct mechanistic link between increased goblet cell density and gut barrier dysfunction in type 1 diabetes. Second, it highlights the utility of intravital multiphoton microscopy for the real-time evaluation of barrier function and therapeutic interventions [35,36]. Third, it raises the possibility that modulating goblet cell biology could complement existing strategies for infection prevention and metabolic control in patients with type 1 diabetes.

Beyond diabetes, alterations in goblet cell biology and barrier integrity have been implicated in a spectrum of intestinal and systemic disorders. In inflammatory bowel disease, for instance, goblet cell depletion and mucus layer thinning contribute to microbial translocation and chronic inflammation [37], a mechanism distinct from that of goblet cell hyperplasia observed in our diabetic model. Conversely, in obesity and non-alcoholic fatty liver disease, low-grade inflammation and dysbiosis have been associated with impaired barrier function, suggesting that goblet cell-mediated leakage may represent a diabetes-specific phenotype [38,39]. These comparisons underscore the need to contextualise barrier dysfunction within different disease environments.

Clinically, our findings highlight goblet cells as a potential therapeutic target. While prebiotics such as FOS appear promising, future studies should compare their efficacy with that of other interventions, including inulin, galactooligosaccharides, or the direct administration of butyrate and other postbiotics. Understanding whether these strategies differentially modulate goblet cell function may inform the development of microbiota-based therapies for type 1 diabetes and related disorders.

In summary, our results reveal that increased intestinal permeability in type 1 diabetic mice is primarily mediated by goblet cell-associated transcellular leakage rather than increased cell shedding or gap formation. FOS supplementation effectively reverses these changes, indicating its potential as a therapeutic agent for restoring gut barrier integrity in type 1 diabetes. Future studies should extend these findings by examining the microbiota–epithelial–immune axis in greater detail, comparing type 1 and type 2 diabetes models, and evaluating whether long-term prebiotic supplementation can mitigate infection risk and systemic inflammation in clinical settings.

### Limitations

This study has some limitations. First, we employed an STZ-induced mouse model, which primarily reflects the pancreatic β-cell destruction and insulin deficiency characteristic of type 1 diabetes. Therefore, this model does not fully replicate the complex insulin resistance and metabolic dysregulation observed in human type 2 diabetes nor does it capture the full spectrum of long-term diabetic complications. Second, the sample size in each group was relatively small, which may have limited the statistical power of our findings. Third, we only evaluated the short-term effects of FOS supplementation, and its long-term efficacy and safety remain to be determined. Finally, the study did not directly assess the gut microbiota composition or delineate the molecular mechanisms underlying goblet cell regulation.

## 4. Materials and Methods

### 4.1. Animals

A total of 15 male C57BL/6 mice aged 6–8 weeks and weighing 20–25 g were used in this study. Healthy mice were obtained from the National Laboratory Breeding and Research Center, while STZ-induced diabetic mice were sourced from the Laboratory Animal Center of National Sun Yat-sen University. The Animal Ethics Committee of National Taiwan University approved all animal procedures.

### 4.2. Experimental Design

Three groups of mice were studied: Group 1 (control): Healthy mice without diabetes (*n* = 5); Group 2 (STZ): Mice with STZ-induced type 1 diabetes (*n* = 5); and Group 3 (STZ + FOS): STZ-induced type 1 diabetic mice treated with FOS for 1 week (*n* = 5). Intestinal permeability and morphological alterations were evaluated using intravital multiphoton microscopy.

### 4.3. Assessment of Intestinal Permeability

To quantitatively assess intestinal permeability, fluorescein isothiocyanate-labelled dextran (FITC-dextran, molecular weight 4000 Da; FD4; Sigma-Aldrich, St. Louis, MO, USA; and emission 500–550 nm) was used as a paracellular tracer. A 1 cm segment of the distal ileum was clamped at both ends, and 1 mL of phosphate-buffered saline (PBS, 0.1 M) containing 25 mg of FD4 was injected into the isolated loop. After 2 h, approximately 100 µL of blood was collected from the portal vein. The serum was separated by centrifugation at 4 °C for 7 min, and FITC-dextran concentration was quantified with a fluorescence spectrophotometer (F-2000, Hitachi, Tokyo, Japan; excitation 480 nm, emission 520 nm).

### 4.4. Morphological Analysis of the Intestinal Epithelium

Intravital multiphoton microscopy was used to compare intestinal morphology between the control and STZ groups. Following intra-ileal injection of FITC-dextran, mice were intravenously administered Hoechst 33258 (2 mg/kg; nuclear stain; Sigma-Aldrich, St. Louis, MO, USA; and emission 435–485 nm) and Rhodamine 6G (3 mg/kg; cytoplasmic stain; Sigma-Aldrich, MO, USA; and emission 550–630 nm) via jugular catheterisation. All reagents were of analytical grade and freshly prepared in sterile PBS prior to use. The terminal ileum was then examined to visualise villus architecture and epithelial integrity. According to our previous work [15], epithelial gaps were identified as true cell-free regions that were negative for Hoechst 33258 and Rhodamine 6G staining. In contrast, goblet cells were readily distinguished by their positive nuclear and cytoplasmic staining.

### 4.5. FOS Intervention

To assess the therapeutic effect of fructooligosaccharides (Wako Pure Chemical Industries, Osaka, Japan), STZ-induced type 1 diabetic mice were orally administered FOS for 1 week. These mice then underwent similar permeability and morphological evaluations as described above. Intestinal barrier function was examined using the same imaging protocols.

### 4.6. Quantification of Intestinal Permeability

A 1 cm segment of the distal ileum was clamped at both ends using atraumatic vascular clamps. A total of 1 mL PBS (0.1 M) containing 25 mg of FITC-dextran was injected into the isolated segment. The dye diffused through the intestinal wall and entered the portal circulation. After 2 h, blood was collected from the portal vein. A 100 µL blood sample was centrifuged at 4 °C for 7 min, and the supernatant was analysed using a fluorescence spectrophotometer (F-2000, Hitachi; excitation 480 nm, emission 520 nm). The FITC-dextran concentration was quantified based on fluorescence intensity.

### 4.7. Surgical Preparation and Multiphoton Microscopy

Surgical preparation was performed as described in previous studies [19]. Anaesthesia was induced using a mixture of Zoletil and Rompun and maintained with isoflurane inhalation. A PE-10 catheter was inserted into the internal jugular vein. The abdomen was sterilised with 70% ethanol, and a midline incision was made to expose the terminal ileum. A 1 cm segment, located 3 cm proximal to the ileocecal valve, was clamped and injected with FITC-dextran. After 2 h, the segment was longitudinally incised along the vasa recta border, rinsed with 0.1 M PBS, and mounted on a custom imaging chamber. Mice were positioned prone on a round platform under an inverted microscope, and intravenous fluorescent dyes were administered before image acquisition. The ambient temperature was maintained at 23 °C throughout imaging.

**Figure 5:**
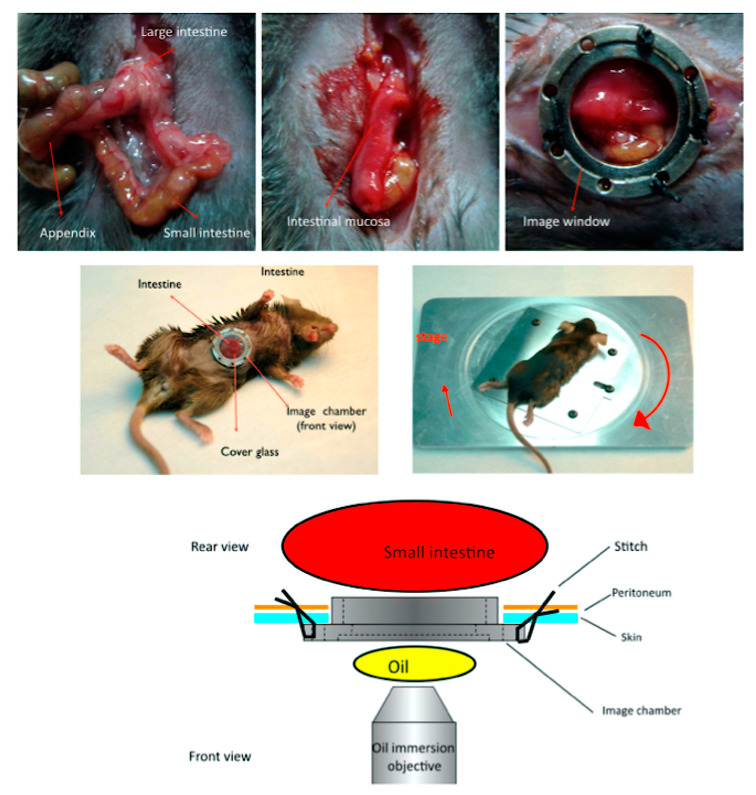
Surgical preparation and setup for intravital multiphoton imaging of the mouse small intestine. Top row: Sequential images showing the exposed mouse intestine, visualisation of the intestinal mucosa, and mounting of the intestine within a custom image window. Middle row: Photographs of the mouse positioned on the imaging platform with the exposed intestine stabilised in the imaging chamber and covered with a glass window. Bottom: Schematic diagram (rear and front views) of the imaging setup. A 1 cm segment of the small intestine is secured within the imaging chamber and imaged through a cover glass using an oil immersion objective. The chamber design allows for stable intravital imaging while maintaining tissue viability. Multiphoton imaging was performed using an inverted Nikon TE2000 microscope equipped with S Fluor 40×/NA 1.3 and Plan Fluor 20×/NA 0.75 MI objectives.

Multiphoton imaging was performed using an inverted microscope (Nikon TE2000, Tokyo, Japan) equipped with two objectives: Nikon S Fluor 40×/NA 1.3 and Nikon Plan Fluor 20×/NA 0.75 MI. FITC-dextran (4000 Da) was used to assess permeability (emission 500–550 nm), Hoechst 33258 (435–485 nm) was used to label the nuclei, and Rhodamine 6G (550–630 nm) was used to stain the cytoplasm. Imaging was performed at a depth of 30–50 μm to optimise visualisation of epithelial structures while minimising signal attenuation. Sequential images were recorded at 1 min intervals from the same field immediately after the transition zone was identified (Figure 5).

### 4.8. Statistical Analysis

All data are expressed as the mean ± standard deviation. Statistical comparisons among groups were performed using one-way analysis of variance, followed by Fisher’s exact test or Student’s *t*-test where appropriate. A *p*-value < 0.05 was considered statistically significant. All analyses were conducted using SPSS software, version 15.0 (IBM Corp., Armonk, NY, USA). Each experimental group consisted of five animals.

## 5. Conclusions

Our findings indicate that increased goblet cell-mediated leakage is a major contributor to gut barrier dysfunction in diabetic mice and that FOS supplementation can partially restore barrier integrity. Targeting goblet cell function may be a promising approach for improving gut health in diabetes.

## Data Availability

All data generated and analysed during this study are included in this published article. The raw datasets used and/or analysed during the current study are available from the corresponding author on reasonable request.

## References

[1] Moens E., Veldhoen M. (2012). Epithelial barrier biology: Good fences make good neighbours. Immunology.

[2] Harris C.E., Griffiths R.D., Freestone N., Billington D., Atherton S.T., Macmillan R.R. (1992). Intestinal permeability in the critically ill. Intensive Care Med..

[3] Gatt M., Reddy B.S., MacFie J. (2007). Review article: Bacterial translocation in the critically ill—Evidence and methods of prevention. Aliment. Pharmacol. Ther..

[4] Fry D.E. (2012). Sepsis, systemic inflammatory response, and multiple organ dysfunction: The mystery continues. Am. Surg..

[5] Puleo F., Arvanitakis M., Van Gossum A., Preiser J.C. (2011). Gut failure in the ICU. Semin. Respir. Crit. Care Med..

[6] Samel S., Keese M., Kleczka M., Lanig S., Gretz N., Hafner M., Sturm J., Post S. (2002). Microscopy of bacterial translocation during small bowel obstruction and ischemia in vivo—A new animal model. BMC Surg..

[7] Sima C., Rhourida K., Van Dyke T.E., Gyurko R. (2010). Type 1 diabetes predisposes to enhanced gingival leukocyte margination and macromolecule extravasation in vivo. J. Periodont. Res..

[8] Bosi E., Molteni L., Radaelli M.G., Folini L., Fermo I., Bazzigaluppi E., Piemonti L., Pastore M.R., Paroni R. (2006). Increased intestinal permeability precedes clinical onset of type 1 diabetes. Diabetologia.

[9] Neu J., Reverte C.M., Mackey A.D., Liboni K., Tuhacek-Tenace L.M., Hatch M., Li N., Caicedo R.A., Schatz D.A., Atkinson M. (2005). Changes in intestinal morphology and permeability in the biobreeding rat before the onset of type 1 diabetes. J. Pediatr. Gastroenterol. Nutr..

[10] Guo X., Jin M., Yang M., Liu K., Li J.W. (2013). Type 2 diabetes mellitus and the risk of hepatitis C virus infection: A systematic review. Sci. Rep..

[11] Wang H.H., Tsai S.H., Yu C.Y., Hsu H.H., Liu C.H., Lin J.C., Huang G.S., Cheng W.T., Tung H.J., Chen C.Y. (2014). The association of haemoglobin A_1_C levels with the clinical and CT characteristics of Klebsiella pneumoniae liver abscesses in patients with diabetes mellitus. Eur. Radiol..

[12] Lin Y.T., Wang F.D., Wu P.F., Fung C.P. (2013). Klebsiella pneumoniae liver abscess in diabetic patients: Association of glycemic control with the clinical characteristics. BMC Infect. Dis..

[13] Diamant M., Blaak E.E., de Vos W.M. (2011). Do nutrient–gut–microbiota interactions play a role in human obesity, insulin resistance and type 2 diabetes?. Obes. Rev..

[14] Pickup J.C. (2004). Inflammation and activated innate immunity in the pathogenesis of type 2 diabetes. Diabetes Care.

[15] Lai C.W., Sun T.L., Lo W., Tang Z.H., Wu S., Chang Y.J., Wu C.C., Yu S.C., Dong C.Y., Chen L.W. (2013). Shedding-induced gap formation contributes to gut barrier dysfunction in endotoxemia. J. Trauma Acute Care Surg..

[16] de Kort S., Keszthelyi D., Masclee A.A. (2011). Leaky gut and diabetes mellitus: What is the link?. Obes. Rev..

[17] Fasano A. (2012). Leaky gut and autoimmune diseases. Clin. Rev. Allergy Immunol..

[18] Vaarala O., Atkinson M.A., Neu J. (2008). The “perfect storm” for type 1 diabetes: The complex interplay between intestinal microbiota, gut permeability, and mucosal immunity. Diabetes.

[19] Visser J.T., Lammers K., Hoogendijk A., Boer M.W., Brugman S., Beijer-Liefers S., Zandvoort A., Harmsen H., Welling G., Stellaard F. (2010). Restoration of impaired intestinal barrier function by the hydrolysed casein diet contributes to the prevention of type 1 diabetes in the diabetes-prone BioBreeding rat. Diabetologia.

[20] Vaarala O. (2011). The gut as a regulator of early inflammation in type 1 diabetes. Curr. Opin. Endocrinol. Diabetes Obes..

[21] Cani P.D., Possemiers S., Van de Wiele T., Guiot Y., Everard A., Rottier O., Geurts L., Naslain D., Neyrinck A., Lambert D.M. (2009). Changes in gut microbiota control inflammation in obese mice through a mechanism involving GLP-2-driven improvement of gut permeability. Gut.

[22] Cani P.D., Neyrinck A.M., Fava F., Knauf C., Burcelin R.G., Tuohy K.M., Gibson G.R., Delzenne N.M. (2007). Selective increases of bifidobacteria in gut microflora improve high-fat-diet-induced diabetes in mice through a mechanism associated with endotoxaemia. Diabetologia.

[23] Kleessen B., Hartmann L., Blaut M. (2003). Fructans in the diet cause alterations of intestinal mucosal architecture, released mucins and mucosa-associated bifidobacteria in gnotobiotic rats. Br. J. Nutr..

[24] Cani P.D., Daubioul C.A., Reusens B., Remacle C., Catillon G., Delzenne N.M. (2005). Involvement of endogenous glucagon-like peptide-1 (7–36) amide on glycaemia-lowering effect of oligofructose in streptozotocin-treated rats. J. Endocrinol..

[25] Gomes A.C., Bueno A.A., de Souza R.G.M., Mota J.F. (2014). Gut microbiota, probiotics and diabetes. Nutr. J..

[26] Arrieta M.C., Bistritz L., Meddings J.B. (2006). Alterations in intestinal permeability. Gut.

[27] Cani P.D., Bibiloni R., Knauf C., Waget A., Neyrinck A.M., Delzenne N.M., Burcelin R. (2008). Changes in gut microbiota control metabolic endotoxemia-induced inflammation in high-fat diet–induced obesity and diabetes in mice. Diabetes.

[28] Sapone A., de Magistris L., Pietzak M., Clemente M.G., Tripathi A., Cucca F., Lampis R., Kryszak D., Cartenì M., Generoso M. (2006). Zonulin upregulation is associated with increased gut permeability in subjects with type 1 diabetes and their relatives. Diabetes.

[29] Patel K.K., Miyoshi H., Beatty W.L., Head R.D., Malvin N.P., Cadwell K., Guan J.L., Saitoh T., Akira S., Seglen P.O. (2013). Autophagy proteins control goblet cell function by potentiating reactive oxygen species production. EMBO J..

[30] McDole J.R., Wheeler L.W., McDonald K.G., Wang B., Konjufca V., Knoop K.A., Newberry R.D., Miller M.J. (2012). Goblet cells deliver luminal antigen to CD103+ dendritic cells in the small intestine. Nature.

[31] Balzan S., de Almeida Quadros C., de Cleva R., Zilberstein B., Cecconello I. (2007). Bacterial translocation: Overview of mechanisms and clinical impact. J. Gastroenterol. Hepatol..

[32] Gyurko R., Siqueira C.C., Caldon N., Gao L., Kantarci A., Van Dyke T.E. (2006). Chronic hyperglycemia predisposes to exaggerated inflammatory response and leukocyte dysfunction in Akita mice. J. Immunol..

[33] Han S.H. (1995). Review of hepatic abscess from Klebsiella pneumoniae. An association with diabetes mellitus and septic endophthalmitis. West. J. Med..

[34] Furusawa Y., Obata Y., Fukuda S., Endo T.A., Nakato G., Takahashi D., Nakanishi Y., Uetake C., Kato K., Kato T. (2013). Commensal microbe-derived butyrate induces the differentiation of colonic regulatory T cells. Nature.

[35] Niesner R.A., Hauser A.E. (2011). Recent advances in dynamic intravital multi-photon microscopy. Cytom. Part A.

[36] Weigert R., Sramkova M., Parente L., Amornphimoltham P., Masedunskas A. (2010). Intravital microscopy: A novel tool to study cell biology in living animals. Histochem. Cell Biol..

[37] Johansson M.E.V., Gustafsson J.K., Sjöberg K.E., Petersson J., Holm L., Sjövall H., Hansson G.C. (2010). Bacteria penetrate the inner mucus layer before inflammation in the dextran sulfate colitis model. PLoS ONE.

[38] Lam Y.Y., Ha C.W.Y., Campbell C.R., Mitchell A.J., Dinudom A., Oscarsson J., Cook D.I., Hunt N.H., Caterson I.D., Holmes A.J. (2012). Increased gut permeability and microbiota change associate with mesenteric fat inflammation and metabolic dysfunction in diet-induced obese mice. PLoS ONE.

[39] Miele L., Valenza V., La Torre G., Montalto M., Cammarota G., Ricci R., Mascianà R., Forgione A., Gabrieli M.L., Perotti G. (2009). Increased intestinal permeability and tight junction alterations in nonalcoholic fatty liver disease. Hepatology.

